# Genetic diversity and phylogenetic relationships of the rock ptarmigan (*Lagopus muta*) in Japan based on mitochondrial genome analysis of museum specimens

**DOI:** 10.1371/journal.pone.0339266

**Published:** 2026-02-06

**Authors:** Nobuaki Nagata, Norimasa Sugita, Kazuto Kawakami, Isao Nishiumi

**Affiliations:** 1 Department of Anthropology, National Museum of Nature and Science, Ibaraki, Japan; 2 Donated Fund Laboratory of Conservation Science, National Museum of Nature and Science, Ibaraki, Japan; 3 Department of Botany, National Museum of Nature and Science, Ibaraki, Japan; 4 Animal Linguistics Groups, Research Center for Advanced Science and Technology, The University of Tokyo, Tokyo, Japan; 5 Hokkaido Research Center, Forestry and Forest Products Research Institute, Hokkaido, Japan; 6 Department of Zoology, National Museum of Nature and Science, Ibaraki, Japan; University of the Faroe Islands: Frodskaparsetur Foroya, FAROE ISLANDS

## Abstract

Relict species are important targets for biodiversity conservation and biogeographical research. The rock ptarmigan, *Lagopus muta*, which is distributed across the circumpolar region, is also found as relict populations in high mountain areas of mid-latitude regions worldwide. In Japan, isolated populations occur in the high mountain areas of Honshu Island. The Japanese *L. muta japonica* is threatened with extinction due to its fragmentation into multiple distribution areas, including regions where populations have already become extinct. To clarify the phylogenetic origin of the Japanese population, we conducted a mitochondrial genome-based phylogenetic analysis using museum specimens, including samples that are over 100 years old. A literature survey of the distribution areas near Japan suggested that the northern Kuril Islands represent the closest distribution area, although an alternative theory proposes that Primorsky Krai, on the eastern edge of the Eurasian continent, is nearest. Although the extracted DNA fragments were very short, we successfully determined the complete mitochondrial genome for all 18 individuals. Among the various mitochondrial regions, the control region and *ND5* exhibited the greatest diversity. Network analysis of the control region from 209 individuals across all distribution areas worldwide indicated that the Japanese population is endemic, whereas no clear regional differentiation within Japan was observed in either the control region or the entire mitochondrial genome. Divergence time estimation suggests that the Japanese population diverged approximately 80,000–120,000 years ago, before the Last Glacial Maximum. This study demonstrates that useful genetic information can be recovered from degraded DNA even from specimens stored under high-temperature and high-humidity conditions in Japan, and provides a basis for re-evaluating the evolutionary history and regional genetic independence of *L. muta*.

## Introduction

**Fig 1:**
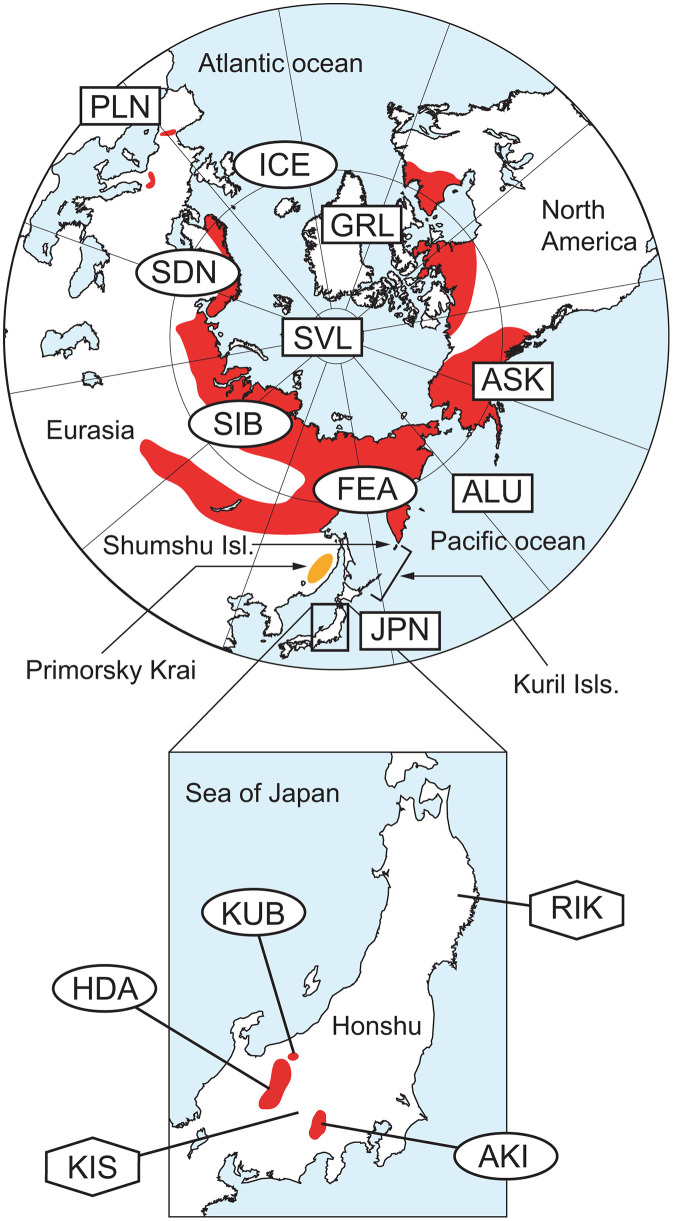
Distribution areas and analysis sites for *Lagopus muta.* The red area indicates the current distribution area. Symbols indicate the source and type of mitochondrial data: Circles denote regions where complete mitochondrial genomes were determined in this study or obtained from databases; Hexagons represent extinct regions for which complete mitochondrial genomes were determined; Squares indicate regions from which only a mitochondrial control region was available via database sources. Three-letter abbreviations are used to designate each region: PLN: Pyrenees; ICE: Iceland; GRL: Greenland; SVL: Svalbard; SDN: Scandinavia; SIB: Siberia; FEA: Far East Asia; ALU: Aleutian; ASK: Alaska; JPN: Japan. In Japan: HDA: Hida Mountains; KUB: Kubiki region; AKI: Akaishi Mountains; RIK: Rikuchu; KIS: Kiso Mountains. The map was generated by the authors using GMT v6.0 [29] with the ETOPO1 global relief model [30], which is in the public domain.

The current distribution of living organisms has been greatly influenced by climate changes during the Pleistocene [1–3]. In particular, organisms inhabiting the cold zones of the Northern Hemisphere have experienced expansions and contractions of their distribution areas in accordance with glacial and interglacial cycles. In the temperate zone, organisms from colder regions expanded their distributions southwards during glacial periods but contracted their distribution areas during interglacial periods. In some cases, these organisms were left behind in refugia such as high mountains, and some species still inhabit these warm refugial areas as relict species [4–6]. These relict species provide valuable opportunities for understanding how organisms respond to climate change. Moreover, due to the limited habitats of relict species, many are now threatened with extinction as a result of habitat destruction and the reduction of inhabitable areas caused by global warming since the 20th century. Rock ptarmigan, *Lagopus muta,* is a bird that is widely distributed, primarily within the Arctic Circle, but it is also found in isolated populations in the temperate zone, such as the European Alps, the Pyrenees, the Pamir Mountains, and Japan, serving as a relict species from the Ice Age (Fig 1). Although the species as a whole is classified as “Least Concern (LC)” on the IUCN Red List [7], it is considered to be more endangered in isolated regions such as Europe and Japan. *Lagopus muta* exhibits morphological variation and comprises multiple known subspecies, prompting extensive genetic research [8–13]. In the Pyrenees, where it is distributed in isolation, it is known to be genetically related to the geographically close populations in Scandinavia [9], and it has become further subdivided within the Pyrenees [14].

In central Honshu, the main island of Japan, there is the central mountainous region that includes the Hida Mountains and Akaishi Mountains, which boast peaks exceeding 3,000 meters, as well as the somewhat lower Kiso Mountains [15]. The Hida Mountains contain small glaciers, and several areas across these ranges still maintain perennial snow, creating a cold environment [16]. As a result, many relict species, such as the alpine butterflies *Anthocharis cardamines, Oeneis norna* and *Aporia hippia,* and the alpine plants *Pedicularis japonica* and *Dryas octopetala*, are distributed in this region. Some of these species are known to be highly genetically differentiated from conspecific populations outside Japan [6,17–21]. *Lagopus muta* populations in Japan are considered to be the endemic subspecies *Lagopus muta japonica*, and currently restricted to the high mountain regions of the Akaishi Mountains and Hida Mountains (including the neighboring independent peaks of Mount Norikura and Ontake), both of which exceed 3,000 meters in elevation, and to the Kubiki mountain range, which ranges from 2,000–2,500 meters and is located to the northeast of the Hida Mountains [22] (Fig 1). They nest in *Pinus pumila* shrubs [22,23] and mainly feed on plants such as *Vaccinium ovalifolium* and *Empetrum nigrum* [22,24]. In the past, they were also distributed in Mount Hakusan, the Kiso Mountains, and Mount Yatsugatake, which ranges from 2,500 to less than 3,000 meters, but these populations are now extinct [22,25]. It is also considered that *L. muta* was historically distributed over a wider area, but its range has since contracted to two primary areas: the northern distribution area, comprising the Hida Mountains and surrounding regions, and the southern distribution area, comprising the Akaishi Mountains. Furthermore, even within its current distribution areas, ecological surveys have reported habitat contraction and a corresponding decrease in population size [22,25]. In the 1980s, the total population was estimated to be around 3,000 individuals, with approximately 2,200 in the Hida Mountains and surrounding areas, and around 800 in the Akaishi Mountains. However, recent surveys have documented a significant decline, with some mountains experiencing a reduction of nearly 80% [22]. This decline has been attributed to habitat loss due to global warming, vegetation degradation caused by the increasing population of sika deer (*Cervus nippon*), and the expansion of natural predators, and there is concern that this trend will persist into the future [22,26]. For these reasons, *L. muta* in Japan is designated as Endangered (EN) under the Japanese Ministry of the Environment’s Red List, despite being classified as LC globally on the IUCN Red List. Although several studies have examined the genetics of the Japanese *L. muta* [27,28], none have comprehensively included the current distribution areas, and it remains unclear when the species first colonized Japan.

Recent advances in next-generation sequencing have made it possible to analyze the mitochondrial and nuclear genomes of avian specimens that have been stored for decades or longer without specific DNA preservation protocols [31–33]. Analyses of museum specimens allow us to study extinct populations without impacting living individuals, making them valuable for advancing the genetics of *L. muta* in Japan. Recently, specimens with labels from Mount Nishi-Koma (Kiso Komagatake), part of the Kiso Mountains and an extinct habitat; specimens thought to be from the Kiso Mountains; and specimens with labels from Rikuchu (an old name for the Tohoku region, whose existence as a distribution area was previously uncertain based on old documents and ambiguous observation reports) were found [34]. We therefore attempted to analyze these specimens to clarify their genetic characteristics, and to provide insights into the genetic diversity and historical biogeography of Japanese *Lagopus muta*.

## Materials and methods

### Specimens and distribution data

*Lagopus muta* has several relict and localized distribution ranges outside the Arctic Circle, although the evidence for the Primorsky Krai range is unclear. In the area around Japan, multiple specimens and other sources have confirmed that *L. muta* is continuously distributed from the Arctic Circle through the northern Kuril Islands and Siberia. On the other hand, the mountainous region of Primorsky Krai, located across the Sea of Japan from Japan, is sometimes considered a part of the species’ distribution range based on historical documents and other sources. However, in many cases, this area is not recognized as part of the distribution range due to insufficient evidence. To clarify whether Primorsky Krai represents a distribution area for *L. muta*, we conducted a literature review and analyzed data from GBIF and other sources.

We analyzed the mitochondrial genome of a total of 18 specimens (Table 1). All specimens analyzed in this study were obtained from the collections of the National Museum of Nature and Science (NMNS) and the Forestry and Forest Products Research Institute. The analyses were performed on preserved specimens that had been deposited in these institutions. No new field sampling was conducted; therefore, no collection permits or site access permissions were required. These included 11 specimens from the Hida Mountains, one specimen from the Akaishi Mountains, one individual from the Kubiki Range, three specimens thought to originate from extinct populations, and two specimens from outside Japan. From the Hida Mountains, we analyzed 10 specimens collected between 1890 and 1954 and one specimen of unknown age. Additionally, we analyzed a portion of frozen muscle collected in 2010 from an individual in the Kubiki Range. All these specimens are stored at the NMNS. We also analyzed one specimen collected in 1953 from the Akaishi Mountains, which is stored at the Forestry and Forest Products Research Institute. Furthermore, three specimens thought to originate from extinct populations were analyzed. These included: a specimen labeled as being collected from Rikuchu in the Tohoku region in 1905 [34], which is stored at the Ehime Prefectural Science Museum; a specimen labeled as being collected from Mount Kiso Komagatake in 1922, which belongs to the Kiso Mountains and is stored at Miyada Elementary School (Miyada Village, Kamiina-gun, Nagano Prefecture, Japan); and a specimen of unknown age, possibly originating from the Kiso Mountains, which is also stored at Komagane Local Museum, Kyodo-kan (Komagane city, Nagano Prefecture, Japan). Lastly, we analyzed two specimens from outside Japan: one collected from Shumshu Island in the northern Kuril Islands in 1931, and another collected in Scandinavia in 2002. Both specimens are stored at the NMNS. Tissue samples were collected from the toe pads of all specimens, except for the individual from the Kubiki Range, from which frozen muscle was analyzed.

**Table 1:** Voucher number, region, locality, capture date, extracted DNA peak size, average extracted DNA size, detected mitochondrial genome size, haplotype of control region.

Voucher number	region	locality	capture date	Peak size	Average size	mitogenome size	haplotype of CR
NSMT-A10067	Hida Mountains	Mt. Norikura	1933	142	196	16686	Type 01
NSMT-A10068	Hida Mountains	Mt. Norikura	1933	110	147	16689	Type 01
NSMT-A10069	Hida Mountains	Mt. Jonen	1915	132	175	16688	Type 01
NSMT-A12486	Hida Mountains	Mt. Yari	1920	121	159	16689	Type 02
NSMT-A12487	Hida Mountains	Mt. Jonen	1890	85	118	16689	Type 02
NSMT-A12488	Hida Mountains	Mt. Tate	1926	70	105	16689	Type 01
NSMT-A12489	Hida Mountains	Mt. Tate	1926	97	142	16688	Type 01
NSMT-A12490	Hida Mountains	Mt. Yari	1919	60	91	16689	Type 01
NSMT-A12491	Hida Mountains	Mt. Yari	1919	91	132	16689	Type 01
NSMT-A12492	Hida Mountains	Mt. Yari	1920	87	122	16689	Type 02
NSMT-A12493	Hida Mountains	Mt. Norikura	?	73	100	16688	Type 01
NSMT-A17980	Kubiki Region	Mt. Hiuchi	2010	8598	>8000	16685	Type 01
NSMT-DNA55123	Kiso Mountains?	?	?	133	208	16689	Type 02
NSMT-DNA55122	Kiso Mountains	Mt. Kisokoma	1922?	94	128	16689	Type 01
FFPRI-2084	Akaishi Mountains	Mt. Kita	1954	132	209	16689	Type 02
NSMT-DNA55124	Rikuchu	?	Dec-1905	92	136	16689	Type 01
NSMT-A20645	Kuril Islands	Shumshu Isl.	14-Aug-1931	93	126	16688	Type 04
NSMT-A21740	Scandinavian Peninsula	Norrbottens	1-Feb-2002	235	324	16682	Type 03
from NCBI/DDBJ/EMBL							
LC528834 [22]	Hida Mountains	–	–	–	–	16687	Type 18
KY411593 [26]	Siberia	–	–	–	–	16683	Type 07
KX609785 [27]	Iceland	–	–	–	–	16687	Type 17

“?” indicates that the information for that field is unknown or not available. Voucher numbers with the prefix “NSMT” and “FFPRI” indicate specimens deposited in the National Museum of Nature and Science, and the Forestry and Forest Products Research Institute, respectively.

### DNA extraction and sequencing

Because the sample DNA was limited in quantity and degraded, molecular experiments were conducted in a contamination-free environment at NMNS. The experiments were performed in a clean bench that had been irradiated with UV light prior to use. Before starting the experiments, the autoclavable pipettes were cleaned and sterilized in an autoclave, and other laboratory equipment was decontaminated using DNA Away (Thermo Fisher Scientific, Waltham, MA, USA). All tips used were filter tips, and both the filter tips and tubes were gamma-sterilized. Each tissue sample was incubated at 55ºC for 2 days in a solution containing 20 µL of Proteinase K (ProK), 10 µL of DTT, 5 µL of 8M Urea, and 180 µL of SDS solution (0.1% SDS, 0.01 M EDTA, and 0.01 M Tris-HCl pH 8.0). After incubation, total DNA was extracted using the MiniElute Reaction Cleanup Kit (QIAGEN, Hilden, Germany). The DNA extraction procedure was performed according to the manufacturer’s instructions, with an additional wash step included. Elution was carried out using EB buffer, and 20 µL of eluent was collected twice to obtain a total volume of 40 µL. The obtained DNA was quantified using the Genomic and D1000 ScreenTapes on the TapeStation 4200 (Agilent Technologies, Santa Clara, CA, USA).

Total DNA was repaired for damage, such as nicks, using the FFPE DNA Repair Kit (New England Biolabs, Beverly, MA, USA). A genomic library for next-generation sequencing was then prepared using the NEBNext Library Prep Kit for Illumina (New England Biolabs), following the manufacturer’s instructions. The genomic library was quantified using the D1000 ScreenTape and TapeStation 4200, and sequencing was performed in 150 bp paired-end mode using the MiSeq with the MiSeq Reagent Kit v2 (300 cycles) (Illumina, San Diego, CA, USA). The quality of the resulting FASTQ files was assessed using fastp [35], and the mitochondrial genome was assembled using two methods: one with GetOrganelle [36] and the other with CLC Genomics Workbench (QIAGEN). The latter was used to perform reference mapping against mitochondrial genomes of individuals from the Japanese Hida Mountain Range (LC528834 [28]), Siberia (KY411593 [37]), and Iceland(KX609785 [38]), which are registered in public databases. These mitochondrial genomes were visually compared with reference sequences and annotated manually based on gene boundaries reported in the reference genomes.

### Data analysis

A total of 21 individuals (18 newly detected samples and 3 database samples) were used to construct a network using the statistical parsimony method (TCS) in PopArt [39]. The analysis included 17 regions in total: 13 protein-coding genes (PCGs), two rRNAs, and the combined sequence of the control region (CR) and integrated tRNAs. Indels were excluded from these analyses. Each protein-coding gene, rRNA, tRNA, and CR was individually aligned using MAFFT v7.526 [40]. To elucidate the phylogenetic position and genetic diversity of the Japanese *L. muta* in a global context, haplotype and nucleotide diversities were calculated using Arlequin ver 3.5.2.2 [41], and the TCS haplotype network was constructed with PopArt based on CR, which provides the most abundant data for *L. muta*. In addition to the 21 individuals whose mitochondrial genomes were analyzed, we obtained CR data for 188 individuals from GenBank, DDBJ, and EMBL [8,9,11,12,42,43]. Based on these 209 individuals, 10 regions were defined: Japan (JPN), Pyrenees (PLN), Scandinavia (SDN), Iceland (ICE), Svalbard (SVL), Greenland (GRL), Siberia (SIB), Far East Asia (FEA), the Aleutian Islands (ALU), and Alaska (ASK) (Fig 1 and S1 Table).

We used the mitochondrial genome to estimate the divergence time of Japanese *Lagopus muta* using BEAST v2.6.7 [44]. In addition to the 18 individuals for which the mitochondrial genome was newly determined and three individuals from public databases, *Lagopus lagopus* (KX609784 [38]) and *Tetrao parvirostris* (MK820678) were included as outgroups. We partitioned the alignment into 17 regions (13 protein‐coding genes, 12S, 16S, concatenated tRNAs, and the control region, CR) and applied uncorrelated lognormal (UCLN) relaxed clocks to each partition under a Yule tree prior. The Bayesian information criterion (BIC) was used to select the optimal nucleotide substitution models for each partition, using ModelFinder Plus included in IQ-TREE version 2.0.7 [45]. A widely used avian mitochondrial rate for *CYTB* is 2.1% pairwise per million years (MY; 1.05% per lineage) [46], whereas the CR evolutionary rate in *Lagopus muta* relative to its sister species *L. lagopus* has been estimated at 6.54% pairwise per MY (3.27% per lineage) [12]. Therefore, we conducted two analyses with alternative prior settings on locus‐specific clock rates. In the first analysis, only the CR partition received an informative lognormal prior on its clock rate with a mean of 0.0327 substitutions/site/MY (specified as the mean in real space), and clock rates for the remaining partitions were estimated with diffuse priors. In the second analysis, only the *CYTB* partition received an informative lognormal prior with a mean of 0.0105 substitutions/site/MY (mean in real space), and the other partitions were estimated as above. For both analyses, starting values (not fixed) for the mean clock rate were set to 0.005 for 12S, 16S and the concatenated tRNAs, and 0.020 for the other partitions. For the calibrated partition in each analysis, the prior standard deviation was set to 0.30 in real space. Sampling was performed every 1000 steps over 200 million MCMC steps. Posterior probabilities were calculated to assess the reliability of each node. We confirmed that the ESS exceeded 200 using Tracer ver. 1.6. To further evaluate the reliability of the phylogenetic tree, we constructed a maximum likelihood (ML) tree using IQ-TREE [45]. Node support values were calculated using 10,000 ultrafast bootstraps (UFBoot) and 1,000 SH-aLRT replicates.

## Results

### Distribution records of *Lagopus muta* in Primorsky Krai

Our investigation into the distribution of *L. muta* in Primorsky Krai revealed the following findings. Although the IUCN Red List indicates that Primorsky Krai within the species’ distribution range, no specific supporting evidence is provided [7]. Furthermore, two bird identification guides published in Russia [47,48] do not list Primorsky Krai as part of the species’ distribution, nor is it depicted on their distribution maps. Additionally, we found no records of specimens from Primorsky Krai in the literature concerning museum specimen collections [49]. A search of the GBIF database [50] also yielded no specimen records or observational data for Primorsky Krai. However, we did encounter a small number of specimen data points plotted outside the known distribution range in the Japanese archipelago; these discrepancies likely result from errors in converting the place names on the labels into latitude and longitude coordinates (the place names corresponded to the current distribution area, but the recorded coordinates were incorrect).

### Mitochondrial genome analysis from specimens

We extracted DNA from a total of 13 specimens collected in Japan before 1940 (stored for 70 to over 100 years). The DNA fragments ranged in peak size from 60 bp to 142 bp, with average fragment sizes between 91 bp and 196 bp (Table 1). In contrast, DNA extracted from frozen tissue showed peak and average fragment sizes exceeding 8,000 bp. The mitochondrial genome was successfully sequenced for all 18 individuals. Sequences from each individual were mapped to three reference genomes, and the resulting alignments were consistent across all three references. In GetOrganelle, the CR was incomplete in some individuals, but the sequences of the 13 PCGs and 2 rRNAs were consistent with the reference mapping in all individuals. The mitochondrial genome lengths ranged from 16,682–16,689 bp. The data have been deposited in the SRA (BioProject PRJNA1231584) and in the NCBI/DDBJ/EMBL databases (accession numbers PV239870-PV239887).

**Fig 2:**
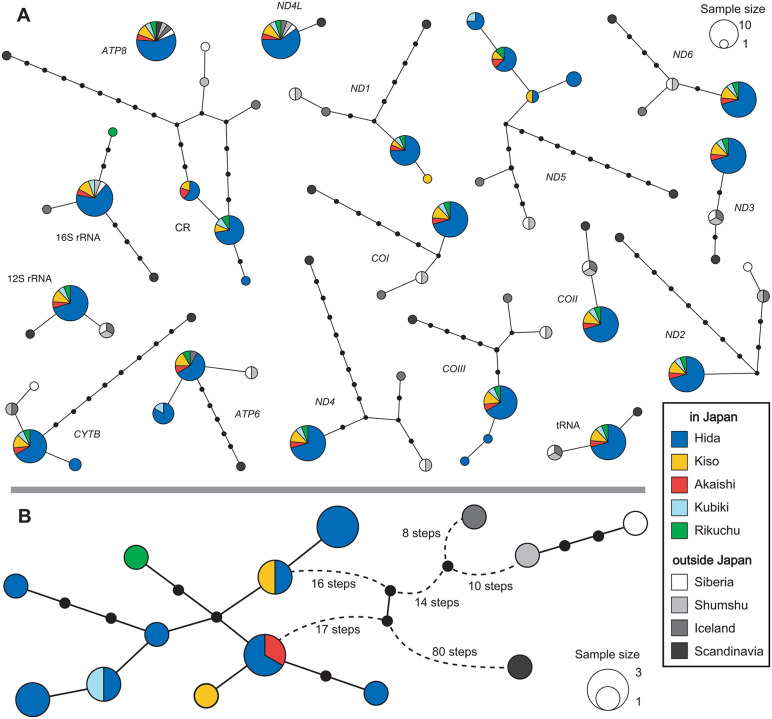
Haplotype networks of the mitochondrial genome. **(A)** Haplotype networks for each of 17 mitochondrial regions (comprising 13 protein‐coding genes, two rRNAs, integrated tRNAs, and the control region). **(B)** Haplotype network for the complete mitochondrial genome of 18 Japanese specimens. The size of each type reflects the number of individuals possessing that haplotype. Small black circles indicate the missing haplotypes.

Among the 17 regions analyzed within the 13 protein-coding genes (PCGs), 2 ribosomal RNAs, the control region (CR), and integrated transfer RNAs (tRNAs) across the 21 individuals (including three from public databases), *ATP6* showed no variation in any region (Fig 2A). *ND4L* showed no mutations within Japan and shared sequences with other regions. Eight regions (*ND6, ND3, ND2, COI, COII, ND4*, tRNA, and 12S rRNA) had no mutations within Japan but differed from sequences in other regions. Five regions (*CYTB, COIII, ND5, ND1*, and CR) displayed variation within Japan, but these variants were not shared with other regions. The 16S rRNA and *ATP6* showed variation within Japan, and some variants were shared with other regions. Overall, the greatest variability was observed in the CR, with 14 steps across 7 types, followed by *ND5* with 13 steps across 7 types. Within Japan, *ND5* exhibited the most variation with 4 types, followed by CR and *COIII*, each with 3 types. Across the whole mitochondrial genome, there were 9 haplotypes in Japan, 5 of which were shared among multiple individuals (Fig 2B).

### Haplotype network, genetic diversity and divergence time analysis

**Fig 3:**
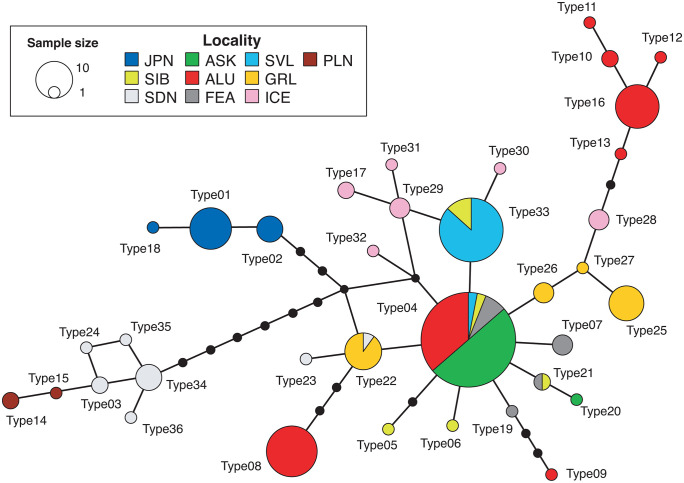
Haplotype network based on partial mitochondrial control region sequence from 209 *Lagopus muta* individuals worldwide. The size of each type reflects the number of individuals possessing that haplotype. Small black circles indicate the missing haplotypes.

Based on 209 individuals from the entire distribution area of the world, there were 36 haplotypes in the network using 1086 bp of partial sequence of CR (Fig 3). Consistent with previous studies [9], Scandinavia and the Pyrenees formed the most divergent clusters. Three distinct haplotypes were identified in Japan, forming a monophyletic group; these haplotypes were unique to Japan and were at least three nucleotide substitutions away from the nearest haplotypes found in other regions. The genetically closest region to Japan was Type 22, identified in Greenland and Scandinavia. However, Type 04, commonly found across the Northern Kuril Islands (Shumshu Island), Aleutian Islands, Siberia, and Alaska, also showed close genetic affinity.

Haplotype diversity (Hd) and nucleotide diversity (π) varied among the 10 regions (Table 2). Hd ranged from 0.0588 (ASK) to 0.8727 (ICE), and π ranged from 0.000108 (ASK) to 0.005157 (SDN). The Japanese *L. muta* (Hd = 0.453, π = 0.000503) showed slightly lower diversity than some regions but was not markedly lower than others (Table 2). These results indicate that the Japanese population does not exhibit a remarkably reduced level of mitochondrial diversity compared to other parts of the species’ range.

**Table 2:** Number of individuals (n), number of haplotypes, haplotype diversity (Hd), and nucleotide diversity (π) of *Lagopus muta* populations from 10 regions based on control region (CR) sequences.

region	n	n of haplotype	Hd	SD	π	SD
ALU	63	8	0.724	0.0282	0.002958	0.001716
ASK	34	2	0.0588	0.0546	0.000108	0.000195
FEA	10	4	0.7111	0.1175	0.000797	0.00069
GRL	22	4	0.6753	0.0574	0.00374	0.002163
ICE	11	6	0.8727	0.0707	0.003947	0.002388
JPN	27	3	0.453	0.0869	0.000503	0.000476
PLN	3	2	0.6667	0.3143	0.000613	0.000765
SDN	12	7	0.8333	0.1002	0.005157	0.002998
SIB	9	5	0.8056	0.1196	0.003373	0.002135
SVL	28	2	0.1376	0.0837	0.000633	0.000552

Values of Hd and π are shown with standard deviations (SD).

**Fig 4:**
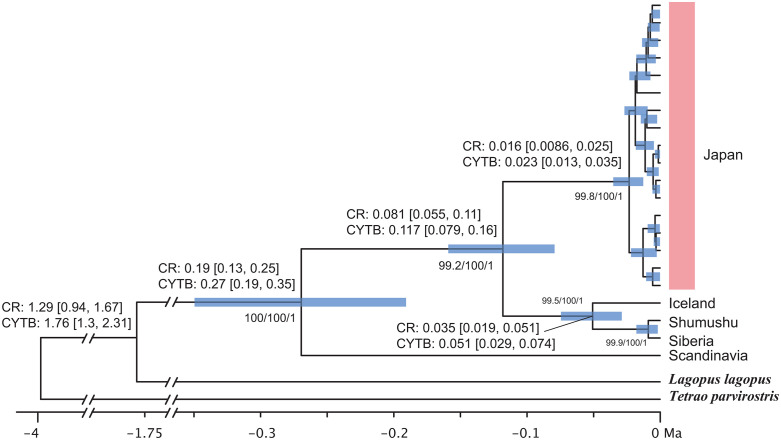
Divergence time estimation based on the mitochondrial genomes of 21 *Lagopus muta* individuals. The tree was time-calibrated using the substitution rate of the *CYTB* gene. Node reliability is indicated by support values from both maximum likelihood (UFboot and SH-aLRT) and Bayesian analyses (Bayesian posterior probabilities); these values are displayed only when UFboot and SH-aLRT values are both ≥ 90 and the posterior probability is ≥ 0.95. For each branch, the divergence times estimated using the CR and *CYTB* rates are shown, with 95% HPD intervals in parentheses.

Divergence time estimates based on the mitochondrial genome of the 21 individuals indicated that the Japanese population formed a well-supported monophyletic group, though no reliable internal nodes were found within Japan (Fig 4). Using two alternative calibrations (*CYTB* – and CR-based), node-age estimates were consistently older under the *CYTB* calibration than under the CR calibration (Fig 3). For the deepest divergence within *L. muta*, the median age was 0.27 Ma (95% HPD 0.19–0.35) under the *CYTB* calibration and 0.19 Ma (95% HPD 0.13–0.25) under the CR calibration. For the Japanese *L. muta* clade, the corresponding estimates were 0.117 Ma (95% HPD 0.079–0.160) and 0.081 Ma (95% HPD 0.055–0.110), respectively (Fig 4).

## Discussion

Worldwide, mitochondrial and whole genome analyses using museum specimens are frequently conducted. Among species that historically inhabited Japan, studies on the mitochondrial and nuclear genomes have been performed for the Japanese river otter (*Lutra lutra nippon*) [51] and the Japanese wolf (*Canis lupus hodophilax*) [52,53]. Research on birds has included the Japanese crested ibis (*Nipponia nippon*) [31] and the Ryukyu wood pigeon (*Columba jouyi*) [33]; however, these specimens are preserved in institutions outside Japan. To our knowledge, no previous study has determined mitochondrial genomes using toepad from long-term preserved bird specimens stored in Japan. In this study, we successfully extracted DNA from 12 bird specimens that had been stored in Japan for over 70 years. The peak DNA fragment length was less than 100 bp, with average fragments ranging from approximately 100–200 bp, indicating a high degree of fragmentation. However, some specimens exhibited longer DNA fragments despite their age. This variation in DNA preservation is likely influenced by storage conditions, including the frequency of fumigation, which accelerates DNA degradation, and fluctuations in temperature and humidity at the storage facility [54]. Despite the challenges posed by DNA degradation, we were able to successfully sequence the whole mitochondrial genomes of all individuals. This result highlights the potential of specimens stored in Japan as valuable resources for genetic research.

The genetic diversity of each mitochondrial gene varied from high to none (Fig 2). Previous studies on genetic diversity in *Lagopus muta i*n Japan have focused on the CR, and our results confirm that the CR exhibits the highest genetic diversity in Japanese populations. *ND5* also exhibited a similar level of variation, suggesting that it is a suitable alternative marker for mitochondrial analyses when additional markers are required beyond CR. The Japanese *Lagopus muta* inhabiting the central mountains region in Honshu represents the southernmost population of this species in East Asia. Although some distribution records (e.g., IUCN Red List) have indicated that *L. muta* also occurs in Primorsky Krai, a region near Japan across the Sea of Japan, our literature survey found no confirmed evidence of its presence there. Based on these findings, it is likely that Primorsky Krai is not part of the current range of *L. muta*. Instead, the closest known distributions to Japan are in inland Siberia and the northern Kuril Islands, approximately 2,000 km away. This geographic distance is nearly equivalent to that between the Pyrenees, the southernmost isolated population in Europe, and the main northern range in Scandinavia, indicating that the Japanese population is geographically as strongly isolated as its European counterparts.

Comparisons of *L. muta* populations worldwide revealed that the Japanese lineage differed from all other populations by at least three substitutions in the partial CR sequence, with a four-base difference even from the most closely related population in the northern Kuril Islands (Shumshu Island; Fig 3). This difference is greater than that observed between populations separated by a comparable geographic distance, such as those in the Pyrenees and the Scandinavian Peninsula. These findings suggest that the Japanese *L. muta* population represents a genetically highly isolated lineage possessing unique evolutionary characteristics distinct from other regions.

Although the comparison is limited to the CR, the genetic diversity of the Japanese *L. muta* population is not particularly low compared to that of other regions worldwide. This finding suggests that, even within isolated and restricted areas, the Japanese *L. muta* population has maintained a certain level of genetic diversity.

In Japan, *Lagopus muta* is currently divided into two main groups: one centered in the Hida Mountains in the north and another in the Akaishi Mountains in the south. We identified three haplotypes in the CR and ten haplotypes in the whole mitochondrial genome. Notably, individuals from the Akaishi Mountains were not genetically distinct from those in the Hida Mountains, as they shared the same haplotypes in both the CR and the whole mitochondrial genome (Fig 2). Among the three specimens thought to originate from extinct populations, the one presumed to be from Rikuchu exhibited two base substitutions in the 16S rRNA gene, distinguishing it from the other individuals. The two specimens, which are thought to be from the Kiso Mountains, differed from each other by three bases in the mitochondrial genome (one difference in the CR sequence). The specimen labeled “Nishikoma” was identical to the Hida Mountains haplotype. In contrast, the specimen with an uncertain label was only different from the Hida and Akaishi Mountains ranges in terms of one base substitution in *ND1*. Therefore, no clear genetic differences were detected in either the CR sequence or the entire mitochondrial genome within Japan. Since *Lagopus muta* is mainly distributed in the Arctic Circle, it might seem that the Japanese *L. muta* population simply migrated southward to Honshu around the time of the Last Glacial Maximum (approximately 20,000 years ago). Many alpine species inhabit the central mountainous region of Honshu, Japan, but their dispersal processes vary. For alpine plants, four dispersal patterns have been reported: (1) species expanding their distribution from the continent to Japan after the Last Glacial Maximum, (2) species that reached Japan before the Last Glacial Maximum, (3) species that diverged within Japan, and (4) species expanding their distribution from Japan to the continent [55]. The divergence time for Japanese *L. muta* was estimated to be approximately 80,000 years ago based on CR and approximately 120,000 years ago based on *CYTB* (Fig 4). This divergence falls within Marine Isotope Stage 5 (MIS5: approximately 130,000–70,000 years ago) [56], specifically corresponding to the period of cooling following the peak warming of the Last Interglacial (MIS5e; approximately 130,000 years ago), likely during MIS5d–c. Mean annual temperatures during that period were estimated to be roughly 5 °C lower than present [56–60]. Furthermore, based on the branching order in the mitochondrial genome phylogenetic tree (Fig 4) and the structure of the CR haplotype network (Fig 3), the Japanese *L. muta* population is considered to have derived from lineages outside Japan. These findings suggest that *L. muta* likely migrated southward from the Arctic Circle during a cold period preceding the Last Interglacial (probably MIS6), expanding its range to Honshu. Subsequently, the warming during the Last Interglacial period isolated them from other populations, forming their current restricted distribution range. Furthermore, despite the cooling during the Last Glacial period, no gene flow occurred with other extant populations. Thus, the Japanese *L. muta* can be regarded as a relict species corresponding to distribution pattern (2) of alpine plants, the “Pleistocene expansion type,” having advanced into Japan before the Last Glacial Maximum and subsequently persisted in isolation.

## Conclusion

In this study, we analyzed the mitochondrial genomes of historical *Lagopus muta* specimens stored in museums and other institutions. Our results indicate that the Japanese *L. muta* is a highly endemic lineage that diverged approximately 80,000–120,000 years ago, that is, after the Last Interglacial (MIS5e) and before the Last Glacial Maximum. This clearly indicates that the Japanese *L. muta* population is an evolutionarily unique lineage in need of conservation. Importantly, this study is the first to determine mitochondrial genomes from toepad samples of long-term preserved bird specimens stored in Japan, demonstrating that short-read next-generation sequencing is a feasible approach for such historical materials and may also enable whole-genome analyses. Although our mitochondrial genome analysis did not reveal any clear genetic differentiation within the Japanese distribution range, including extinct populations, a preliminary SSR analysis (unpublished) suggests genetic differentiation between populations from the Hida Mountains and those from the Akaishi Mountains. Considering these findings, future research incorporating whole-genome analyses will be essential to further elucidate the phylogenetic relationships among regional populations and extinct lineages of *L. muta* in Japan, and to guide the establishment of ex situ conservation programs and reintroduction areas for each lineage.

## Supporting information

S1 Table List of haplotypes, accession numbers, locality codes, localities, and references used in this study. “Note” column shows haplotype IDs or sample names as described in the original references.
(XLSX)
